# Activated carbon preparation from eucalyptus wood chips using continuous carbonization–steam activation process in a batch intermittent rotary kiln

**DOI:** 10.1038/s41598-021-93249-x

**Published:** 2021-07-06

**Authors:** Sumrit Mopoung, Nuchjira Dejang

**Affiliations:** 1 Chemistry Department, Faculty of Science, Naresuan University, Phitsanulok, 65000 Thailand; 2 Department of Physics, Faculty of Science, Naresuan University, Phitsanulok, 65000 Thailand

**Keywords:** Chemistry, Materials science

## Abstract

The production of activated carbon from eucalyptus wood chips by steam activation in a 2000 kg batch intermittent rotary kiln with continuous carbonization–steam activation process conducted at 500 °C to 700 °C was studied. The activated carbon products were characterized by FTIR, SEM–EDS, Raman spectroscopy, and BET analysis. Percent yields, iodine number, and methylene blue number of the produced activated carbon materials were measured as well. It was shown that the percent yields of the activated carbon materials made in the temperature range from 500 to 700 °C are 21.63 ± 1.52%–31.79 ± 0.70% with capacities of 518–737 mg I_2_/g and 70.11–96.93 mg methylene blue/g. The BET surface area and micropore volume of the activated carbons are 426.8125–870.4732 m^2^/g and 0.102390–0.215473 cm^3^/g, respectively. The steam used in the process could create various oxygen containing surface functional groups such as –CO and –COC groups. In addition, it could also increase the amorphous nature of the activated carbon product. These properties of the activated carbon products are increased with increasing steam activation temperature from 500 to 700 °C. As a result, the activated carbon materials produced at activation temperatures of 600 °C and 700 °C exhibit higher adsorption.

## Introduction

Nowadays, the number of environmental problems in the world is rising. This is especially true for wastewater pollution, which needs to be improved in terms of quality for discharge and consumption. Therefore, there is a need to find new methods and materials to be used in the water treatment process. One of the most widely used materials for the treatment of water is activated carbon, which has good absorbent properties. Furthermore, it is cheap and there are a lot of precursor raw materials suitable for activated carbon production. Biomass is a suitable material for producing good quality activated carbon, which is due to its good structural porosity and the ease of adding further micropores by activation. It is also a precursor material that is continuously made in nature and industry. Wastes from the wood industry are low cost bulk materials. Therefore, these waste materials are likely to be a feasible option to produce activated carbon. Eucalyptus wood waste is an abundant waste material from the wood industry, which is being used in many areas, such as briquette fuel^1^, torrefied solid fuel^2^, sugar, gasoline^3^, or biooil^4^ and electrode materials in supercapacitors^5^. Normally, the activated carbon production has two stages. These are the carbonization and activation stages. After the carbonization step is completed and the char is obtained, the furnace is turned off and the reactor is cooled down to ambient temperature prior to the activation stage. The activation methods for activated carbon production include chemical and physical activation. Steam activation is one method of physical activation used in activated carbon production, which usually operates between 700 and 1000 °C^6^. The production of activated carbon with steam is a low cost, green, viable, and effective environmental remediation tool^7^. The activated carbon from *Miscanthus sacchariflorus* prepared with steam activation at 800 °C is high surface area material with increased density of micropores. This is because steam removes carbon atoms from within the solid carbon network, resulting in the creation of new pores and opening of clogged ones^8^. The pyrolysis reactors, which are most widely used for activated carbon production, can be divided into two types by method and load speed. These are fixed-bed and movable-bed reactors. The movable-bed reactors are further subdivided into pneumatic (bubbling, spouted, circulating, or transport fluidized beds), mechanical (rotary kiln, rake, auger, ablative, stirred) reactors, and reactors in which the charge moves under gravity^9^. For fixed-bed reactors, a 1000 kg scale tubular type fixed bed reactor has been used for activated carbon production from spent mushrooms, which obtained activated carbon product of high quality^7^. However, the rotary kiln reactor was selected for activated carbon production in this research. This is because it has many advantages over other types of reactors such as easily adjusted residence time of solids in the reactor, good mixing of materials, and good heat transfer during slow rotation of the inclined kiln. These properties lead to uniform pyrolytic products, and the possibility of continuous operation, even though the solid input materials may have various shapes, sizes, and calorific values^9^.

In this research, eucalyptus wood chips were used as the precursor material for the production of activated carbon by steam activation on a large scale (2000 kg). The 2000 kg pyrolysis reactor is an intermittent rotary furnace which is developed into a prototype factory for commercial activated carbon production. It is adjusted to operate continuously without temperature reduction after the carbon production stage. When the charcoal production process is complete, steam is introduced immediately to reduce costs and processing time. The effect of steam activation temperature of 500 °C, 600 °C, and 700 °C on the properties of the activated carbon from eucalyptus wood chips was investigated.

## Results and discussion

### Proximate analysis and percent yield

Proximate analysis (Table 1) has indicated that raw eucalyptus wood chips constitute a good biomass for activated carbon production with high fixed carbon content and very low ash content. The yield of the activated carbon has decreased from 31.79 ± 0.70% to 21.63 ± 1.52% when the steam activation temperature was increased from 500 to 700 °C. Concomitantly the volatile matter content has decreased. On the other hand, the fixed carbon content has increased with increasing steam activation temperature. These results are due to the thermal degradation of volatile matter and decomposition of carbon in the form of CO, CO_2_, and CH_4_^10^. Partial oxidation with water vapor, as shown in reactions (1–4), has taken place to a higher degree at higher activation temperatures^11^.

1 $${\text{C}} + {\text{H}}_{2} {\text{O}} \to {\text{CO}} + {\text{H}}_{2}$$

2 $${\text{CO}} + {\text{H}}_{2} {\text{O}} \to {\text{CO}}_{2} + {\text{H}}_{2}$$

3 $${\text{C}} + {\text{CO}}_{2} \to 2{\text{CO}}$$

4 $${\text{C}} + 2{\text{H}}_{2} \to {\text{CH}}_{4}$$

**Table 1:** Percent yield and proximate analysis of eucalyptus wood chips and activated carbon.

Sample	Moisture (wt%)	Volatile matter (wt%)	Ash (wt%)	Fixed carbon (wt%)	%yield
Raw eucalyptus wood chips	7.89 ± 0.22	20.87 ± 0.22	1.93 ± 1.18	68.93 ± 0.09	–
Activated carbon at 500 °C	2.63	5.95	1.59	70.32	31.79 ± 0.70
Activated carbon at 600 °C	1.54	3.2	1.43	79.23	25.84 ± 0.37
Activated carbon at 700 °C	1.16	0.89	1.04	80.54	21.63 ± 1.52

### TGA of eucalyptus wood chips

**Figure 1:**
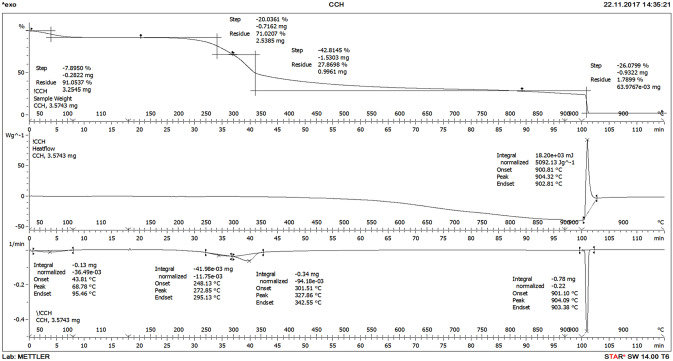
TGA graphs of dried raw eucalyptus wood chips.

Thermal behavior (TGA) of dried raw eucalyptus wood chips was investigated from 40 to 1000 °C at the rate of 10 °C/min in oxidizing atmosphere and the results are shown in Fig. 1. The figure depicts four-stage weight loss. The weight loss during the first stage of TGA, below 200 °C, was found to be 7.90%. This is due to the removal of water content from the cellular structure of eucalyptus wood chips. The second heating stage from 200 to 342 °C resulted in a weight loss of 20.04%, which can be attributed to the decomposition of carbohydrates, cellulose, and hemicellulose components of the eucalyptus wood chips. The hemicelluloses, which consist of an amorphous structure with a low degree of polymerization, were decomposed within a temperature range of 240–295 °C, while cellulose, which is a crystalline material consisting of large chain high molecular-weight-polymer, was decomposed from about 300 to 342 °C in the second stage^12^. The third heating stage from 342 to 900 °C resulted in the weight loss of ~ 69% which is mainly due to the significant decomposition of lignin, which is comprised from a complex aromatic polymer structure, carbonaceous structures, and inorganic impurities. In the last step, beyond 900 °C, there is only a minimal weight loss resulting in residue ash weight of 1.80%. The TGA results are in line with the carbon content of the activated carbon materials obtained after carbonization and steam activation under an inert atmosphere as reported in the proximate analysis results.

### EDS analysis

The results of energy dispersive X-ray analysis (Table 2) show that the raw eucalyptus wood chips contained 51.99% of carbon and 35.35% of oxygen together with other elements such as Na, Si, K, and Ca. After the carbonization-steam activation, the carbon content has increased while the oxygen content has decreased for all activated carbon materials, which were prepared by carbonization-steam activation at 500–700 °C. This is attributed to the removal of volatile matter and oxygen containing functional groups by thermal degradation during the carbonization stage and steam activation stage, respectively. The resulting oxygen content in the steam activated materials was expected to be from oxygen containing functional groups on the surface of the steam activated carbon materials. These results indicate that the steam activated carbon materials exhibit high carbon content and some oxygen containing surface functional groups, which are providing an active surface for the attachment of organic pollutants. Furthermore, the content of certain other elements has also decreased with increasing steam activation temperature. It is assumed that these were more readily dissolved and eluted by steam at higher activation temperatures.

**Table 2:** Elemental composition determined by energy dispersive X-ray spectrometer (EDS).

Samples	Elements composition (wt%)					
	C	O	Na	Si	K	Ca
Eucalyptus wood chips	51.99	35.35	00.61	00.58	03.95	07.35
Activated carbon at 500 °C	73.09	22.95	0.46	0.96	0.43	2.12
Activated carbon at 600 °C	80.52	18.40	0.17	0.28	0.19	0.61
Activated carbon at 700 °C	81.83	17.07	0.10	0.24	0.16	0.60

### FTIR analysis

**Figure 2:**
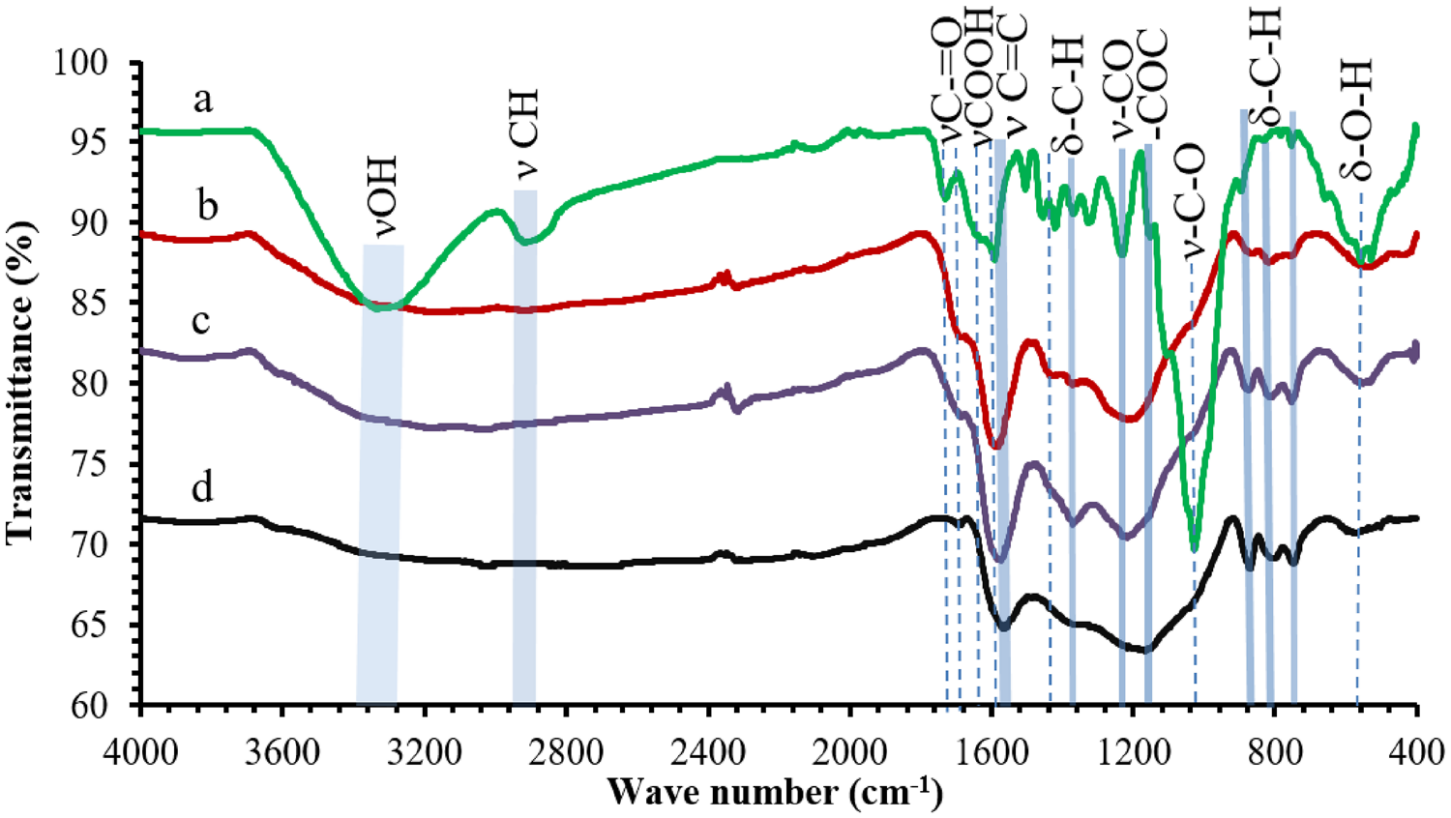
FT-IR transmission spectra of (**a**) dried raw eucalyptus wood chips, (**b**) eucalyptus wood chip activated carbon prepared at 500 °C, (**c**) eucalyptus wood chip activated carbon prepared at 600 °C, and (**d**) eucalyptus wood chip activated carbon prepared at 700 °C.

The infrared transmission spectra of the dried raw eucalyptus wood chips and eucalyptus wood chip activated carbon materials are shown in Fig. 2. The FTIR transmission spectra of eucalyptus wood chips (Fig. 2a) exhibit a number of bands or peaks, which correspond to hydroxyl groups (ν–OH) of carboxylic acids, phenols or alcohols, and adsorbed water (3331.96 cm^−1^). Furthermore, the vibrations corresponding to ν–C–H bonds of methyl and methylene groups can be found at 2918.73 cm^−1^. The vibrations corresponding to ν–C=O in carboxyl (–COOH) or carbonyl groups in ketone, aldehyde, lactone, and carboxyl groups are found at 1730.28 cm^−1^. The vibrations corresponding to ν–C=C– of aromatic rings are found at 1593.21 cm^−1^. The vibrations ν–C=C corresponding to other carbon–carbon unsaturated bonds are found at 1504.88 cm^−1^. The vibrations corresponding to δ–C–H and –C=O of carbonyl and carboxylate ion groups are found at 1454.99 cm^−1^. The vibrations corresponding to –C=O are found at 1324.38 cm^−1^. The vibrations corresponding to ν–CO are found at 1250 cm^−1^. The vibrations corresponding to –COC anti-symmetric bridge are found at 1160 cm^−1^. The vibrations corresponding ν–C–O in acids, alcohols, phenols, ethers, and esters are found at 1029.70 cm^−1^. The vibrations corresponding to δ–C–H are found at 750–898 cm^−1^. Finally, vibrations corresponding to δO–H are found at 580 cm^−1^, respectively^10,13^. The –OH (3331.96 cm^−1^), νC–H bonds (2918.73 cm^−1^), –C=O (1324.38 cm^−1^), –COC anti-symmetric bridge (1160 cm^−1^), ν–C–O (1029.70 cm^−1^), and –C–H (898 cm^−1^) functional groups represent the cellulose structure. Hemicellulose is represented by νC–H bonds (2918.73 cm^−1^), –C=O (1730.28 cm^−1^), –COC anti-symmetric bridge (1160 cm^−1^), and ν–C–O (1029.70 cm^−1^) functional groups. Finally, the νC–H bonds (2918.73 cm^−1^), ν–C=C aromatic vibrations (1593.21 cm^−1^ and 1504.88 cm^−1^), δ–C–H of aliphatic hydrocarbons (1454.99 cm^−1^), ν–CO stretching of acetyl groups (1250 cm^−1^), and –CH groups (750–898 cm^−1^) are components of lignin structure^14^. These bands or peaks found on dried raw eucalyptus wood chips have disappeared after steam activation at temperatures of 500–700 °C. This indicates the thermal degradation of surface functional groups during the carbonization stage, which were decomposed to CO and CO_2_ gasses^10^. The C–H bond and O–H groups were removed from all of the activated carbon materials (Fig. 2b–d) by dehydration effect during the carbonization stage^15^. The steam activated carbon materials prepared at 500–700 °C, spectra of which are shown in Fig. 2b–d, exhibit newly created bands or peaks. It can be seen that the vibration bands, which have maxima located at 1593 cm^−1^ and 1720 cm^−1^, are demonstrating the presence of C=C aromatic ring stretching vibrations and C=O stretching vibrations of carbonyl and carboxylate ion groups^15^, respectively. The presence of C=C aromatic ring stretching vibration, which has shifted from 1593.21 cm^−1^ in raw eucalyptus wood chips (Fig. 2a) to 1504.88 cm^−1^ in the activated carbon materials (Fig. 2b–d), may be attributed to reduction of sidechain aromatic ring-oxygen-containing surface functional groups and high degree of graphitization after carbonization at high temperature^9^. On the other hand, the peak of C=O groups found at 1720 cm^−1^ in raw eucalyptus wood chips has transformed to a weak peak at about 1700 cm^−1^ after activation. Furthermore, the results indicate that C=C aromatic ring stretching vibration tends to increase when the C=O vibration decays more extensively with increasing activation temperature. On the other hand, the weak bands of the –CO group at 1250 cm^−1^ and –COC group at 1160 cm^−1^ have also appeared in the spectra after steam activation, which is due to partial oxidation. In addition, three very weak bands of surface aromatic –CH groups between 750 and 898 cm^−1^ were found on of the activated carbon products after steam activation and tend to increase with increasing activation temperature. This indicates that the aromatic content of the ring structures^16^ has increased with increased temperature of steam activation. The content of carbonyl groups and substitutions in aromatic rings has also increased. The FTIR results show that the activated carbon materials from eucalyptus wood chips prepared with steam activation should be able to interact with polar and non-polar adsorbates by surface functional groups^14^.

### Raman spectroscopy analysis

**Figure 3:**
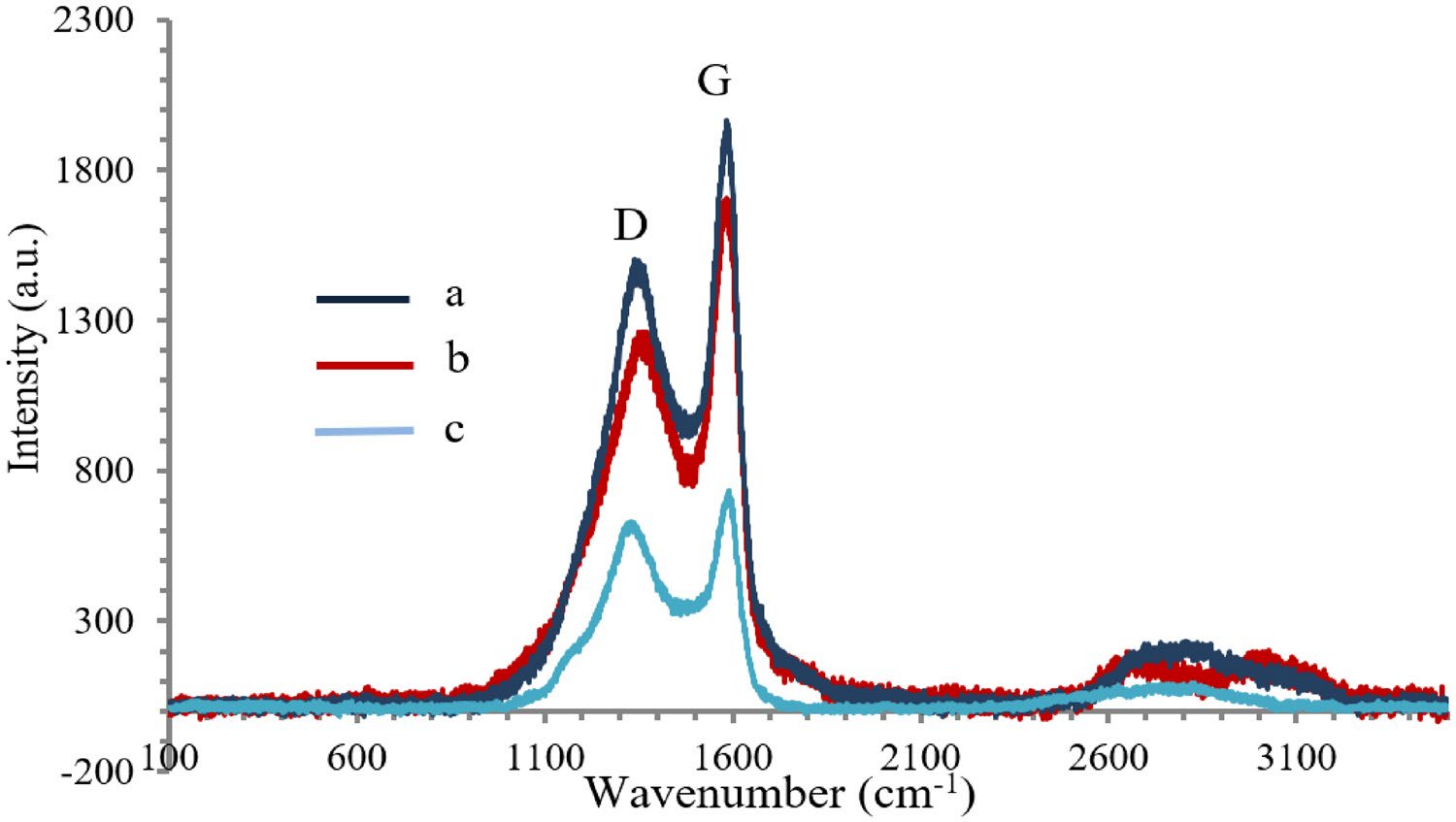
Raman spectra of (**a**) eucalyptus wood chip activated carbon prepared at 500 °C, (**b**) eucalyptus wood chip activated carbon prepared at 600 °C, and (**c**) eucalyptus wood chip activated carbon prepared at 700 °C.

The Raman spectra of eucalyptus wood chips and activated carbon products prepared with steam activation at temperatures of 500–700 °C are shown in Fig. 3. The D band and G band are located at 1350 cm^−1^ and 1587 cm^−1^, respectively. It is known that the D band at 1350 cm^−1^ corresponds to disordered and structurally defected regions consisting of the *sp*^3^-hybridized carbons. On the other hand, the G band at 1587 cm^−1^ corresponds to the stretching of the C=C bond in graphitic carbons with *sp*^2^ hybridized carbon systems^13^. The ID/IG ratio is therefore indicative of amorphous degree of the material. The results indicate that ID/IG ratios increase with increasing activation temperature from 500 to 700 °C. The observed values are 0.74 (500 °C), 0.77 (600 °C), and 0.83 (700 °C). This shows that the surface defects or amorphous degree of the activated carbon products increase with increasing activation temperature, which has been proven to be beneficial for adsorbent applications. This result demonstrates that the surface carbons of eucalyptus wood chip chars were more extensively oxidized by steam (H_2_O) to CO_2_ and CO at higher activation temperatures. The surface porosity of activated carbon has also increased with increasing activation temperature due to the increasing disorder and defects of the carbon structure. This indicates that the steam-activated carbon from eucalyptus wood chips has mixed characteristics of amorphous and graphitic carbons.

### XRD analysis

**Figure 4:**
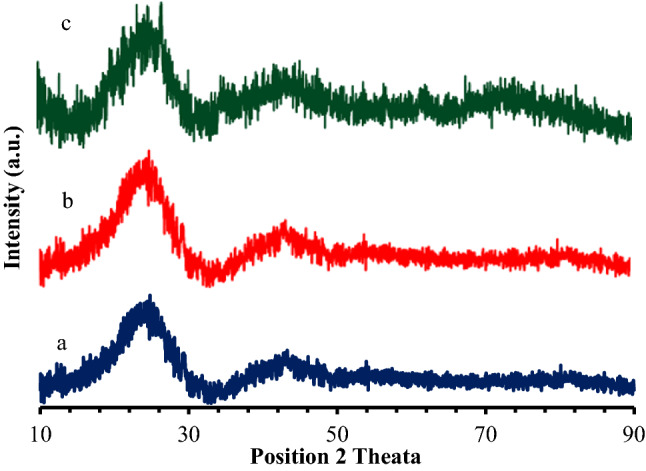
XRD patterns of (**a**) eucalyptus wood chip activated carbon prepared at 500 °C, (**b**) eucalyptus wood chip activated carbon prepared at 600 °C, and (**c**) eucalyptus wood chip activated carbon prepared at 700 °C.

Figure 4 shows the X-ray diffraction pattern of eucalyptus wood chip activated carbon materials prepared at temperatures of 500 °C, 600 °C, and 700 °C. It can be seen that the carbon structure of all activated carbon products is mainly amorphous with the presence of some crystalline material. This is demonstrated with the broad XRD bands at ~ 26° and ~ 44°, respectively, which correspond to an amorphous structure composed of randomly oriented aromatic graphene-like sheets^17^. This is because the steam activation process causes the graphitic structure to be destroyed and the remaining carbon sheets are predominantly disordered with pores on the surface area of steam activated carbon. This result is in good agreement with the Raman spectroscopy results.

### Surface properties of steam activated carbon

Functional groups on the surface of the activated carbon have been shown to have a large effect on the reaction of polar and non-polar substances. On the other hand, the porous structure of the activated carbon governs its adsorption capacity. Therefore, the surface porous properties of activated carbon materials are also an important factor in adsorption. In this study, the surface areas and porosity of the eucalyptus wood chip activated carbon materials resulting from steam activation at different temperatures were investigated and are shown in Table 3. It can be seen that the BET surface area and micropore volume increase with increasing temperature of the steam activation step from 500 to 700 °C. This indicates that increasing the steam activation temperature enhances the development of pores and creates new pores. This is due to more extensive thermal degradation of surface carbon on eucalyptus wood chip char concomitant with partial oxidation by steam as a result of higher activation temperature. This conclusion is supported by the results of Raman and XRD analyses. In addition, it can be seen that the content of pores with dimensions in the range of mesopores and macropores (17 Å and 3000 Å) also increases with increasing steam activation temperature. This may be the result of new micropores being created and existing micropores being enlarged as the walls between pores collapsed by the action of steam, which transformed them to mesopores and macropores. The increase in porosity achieved with increasing steam activation temperature (500–700 °C) is due to the higher rate of diffusion of water molecules into the interior of the char structure, which developed a wide-ranging pore network by partial steam oxidation. In addition, some surface carbons were decomposed to a higher degree by thermal degradation with increasing activation temperature.

**Table 3:** Surface area and porosity properties of steam activated carbon determined by gas adsorption (BET) analyzer.

Activation temperature (ºC)	BET surface area (m^2^/g)	Micropore volume (cm^3^/g)	Surface area of pores between 17 and 3000 Å (m^2^/g)	Volume of pores between 17 and 3000 Å (cm^3^/g)
500	426.8125	0.102390	0.1486	0.002570
600	673.2410	0.148693	27.9665	0.021596
700	870.4732	0.215473	30.8633	0.081921

### Morphology by scanning electron microscope

**Figure 5:**
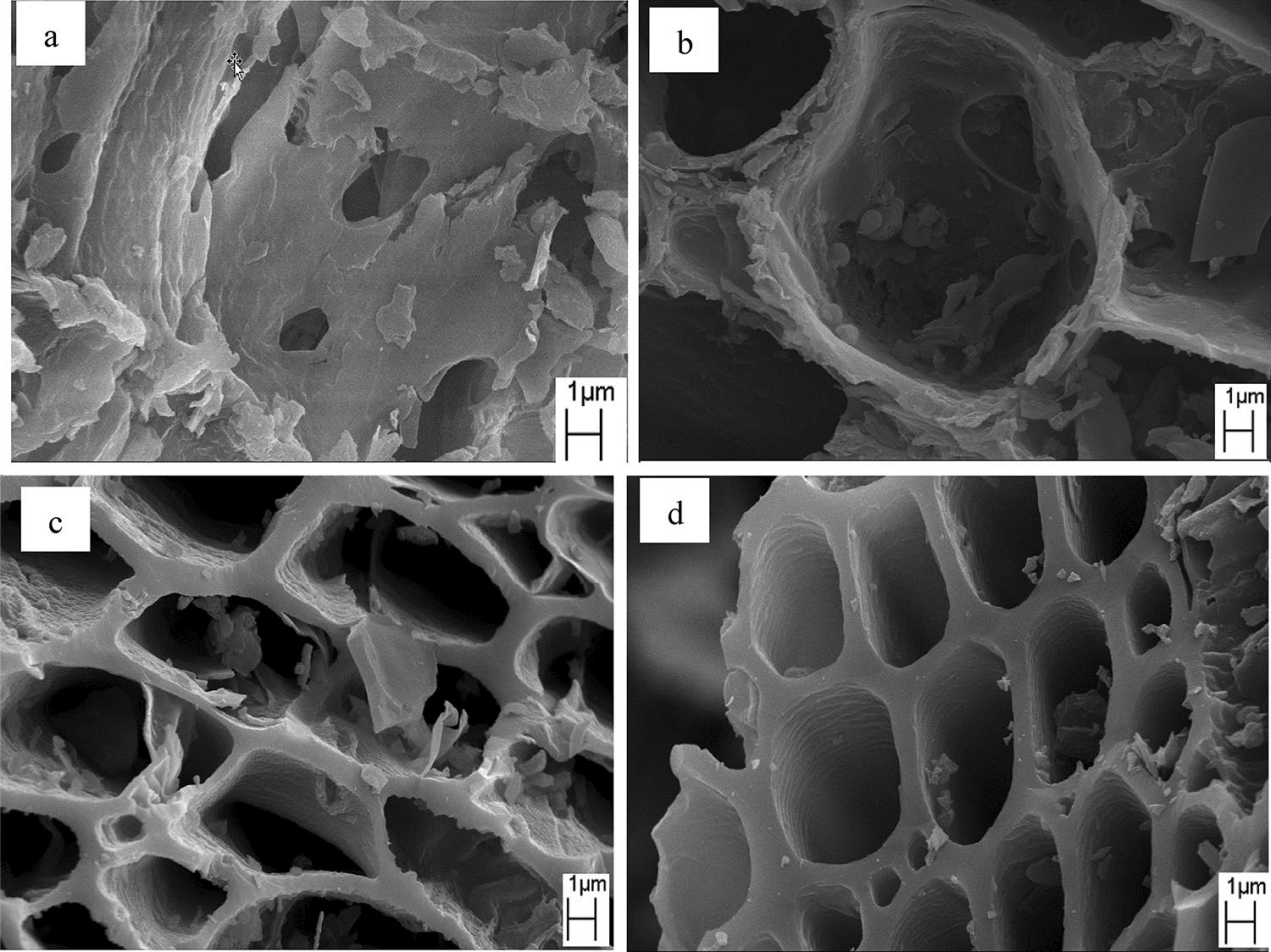
SEM morphology of (**a**) raw dried eucalyptus wood chips, (**b**) eucalyptus wood chip activated carbon prepared at 500 °C, (**c**) eucalyptus wood chip activated carbon prepared at 600 °C, and (**d**) eucalyptus wood chip activated carbon prepared at 700 °C.

The SEM images of raw eucalyptus wood chips and eucalyptus wood chip activated carbon products prepared at 500–700 °C are shown in Fig. 5. For the raw eucalyptus wood chips, the SEM image (Fig. 5a) clearly shows the dense texture, irregular structure with some large pores, and large particles on their surface. However, the activated carbon materials also clearly exhibit large wall-mounted tube structure with very low content of sheet particles after carbonization–steam activation (Fig. 5b–d). Furthermore, it can be seen that the number of sheet particles on the surface of activated carbon products decreases with increasing steam activation temperature from 500 to 700 °C. In the case of activated carbon prepared with steam activation at 500 °C, it can be seen that the tube structures are blocked with a large number of sheet particles. However, the number of sheet particles present on the surface of the carbon material prepared with steam activation at 600 °C is reduced, and the tube structure becomes partially unblocked. In addition, some walls of the tube structure are cracked. Furthermore, the surface of the tube structure of activated carbon prepared with steam activation at 700 °C exhibits a very low amount of sheet particles. This is because ash and disorganized carbon, which formed from decomposition of organic matter and blocked the pores and tube structures during carbonization, were washed out by steam^18^ or burnt out after steam activation. This results in the activated carbon surface being smooth, wrinkled, and exhibiting open holes. These results confirm the results of the investigation of surface properties. In addition, the eucalyptus wood chip steamed activated carbon is mainly composed of tube structures, which should promote the velocity of liquid diffusion for the adsorption process.

### Iodine and methylene blue number of activated carbon

The iodine number and methylene blue number are considered as measures of adsorption capability of activated carbon materials and represent the amount of micropores and mesopores^19^, respectively. In this work, it was observed that all steam activated carbon materials exhibited low methylene blue number in comparison to the iodine number (Table 4). This demonstrates that all the activated carbon materials contain a high amount of micropores with low amount of mesopores, which is suitable for adsorption of small particles and some big organic materials as well. Furthermore, both numbers increase with increasing steam activation temperature. This indicates that the surface area and pore volume of the steam activated carbon materials both increase with increasing steam activation temperature, which is in line with the results of investigation of surface properties and SEM. Thus, it can be concluded that the steam activation contributes to the formation of both micropores and mesopores on the steam activated carbon materials. In this work, the iodine number values of the steam activated carbon materials, which were prepared at the steam activation temperatures of 600 °C and 700 °C, are higher than the value in the Thai industrial standard, which sets the minimal value as 600 mg/g^20^.

**Table 4:** Iodine number and methylene blue index of steamed activated carbon materials.

Activation temperature (°C)	Iodine number (mg/g)	Methylene blue number (mg/g)
500	518	70.11
600	677	85.49
700	737	96.93

## Conclusions

The production of activated carbon from eucalyptus wood chips by steam activation in a 2000 kg batch intermittent rotary kiln with continuous carbonization–steam activation process was investigated. The effect of steam activation temperatures of 500 °C to 700 °C was investigated and it was shown that it could enhance the properties of the activated carbon product for adsorption process. The products exhibit high fixed carbon content and very low ash content. The percent yields of activated carbon materials produced in the activation temperature range of 500 °C to 700 °C are 21.63 ± 1.52%–31.79 ± 0.70% (432.6–635.8 kg), which are sufficient for each batch production. The steam activation has also created some oxygen containing surface functional groups and micropores–mesopores, which provide the active surface for the attachments of the organic pollutants on the surface of the activated carbon product. The activation process also increases the surface areas and pore volumes of the produced carbon materials. In addition, the steam also dissolves ash and disorganized carbon from the activated carbon during steam activation. As a result, the activated carbon materials formed at activation temperatures of 600 °C and 700 °C exhibit a higher iodine number than the value set by the Thai industrial standard, which means that they could be produced commercially. However, the activated carbon products from eucalyptus wood chips made by continuous carbonization–steam activation process in this research need further development to improve their adsorption properties.

## Materials and methods

**Figure 6:**
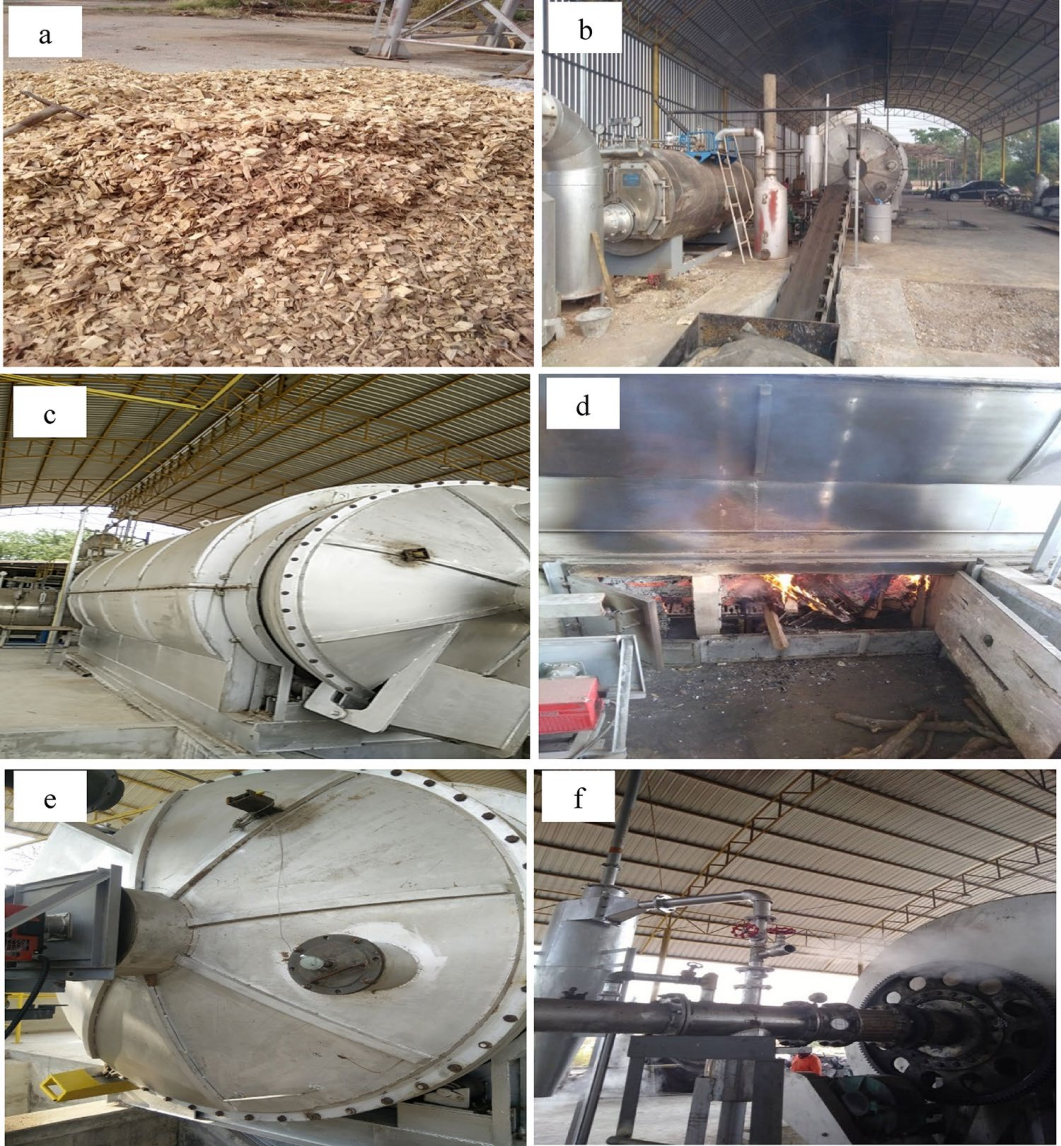
Activated carbon production system of batch intermittent rotary kiln: (**a**) sun dried eucalyptus wood chips, (**b**) conveyor belt, (**c**) rotary kiln, (**d**) wood stove, (**e**) thermocouples, and (**f**) pipe vent (25 mm diameter).

The sun dried eucalyptus wood chips (Fig. 6a) without bark (size 25 mm × 37.5 mm × 5 mm) were collected from a wood factory in Chaiyaphum Province, Thailand. A mass of 2000 kg of eucalyptus wood chips (about 40% of the kiln capacity) was loaded into the rotary kiln (diameter of 2.6 m and a length of 6.6 m (Fig. 6c) by a conveyor belt (Fig. 6b) followed by a fan blowing system. Then, the rotary kiln was closed and wood chips were burned in a wood stove under the rotary kiln (Fig. 6d) with kiln rotation rate of 0.25 rpm. The temperature in the kiln reactor was measured by two thermocouples which were located at the front and end of the kiln (Fig. 6e). The heating rate was 2.5 °C/min until the desired temperature (500 °C, 600 °C, and 700 °C) was reached. After that, continuous steam activation was performed by introducing steam from a boiler (capacity size 1500 kg) into the kiln at a pressure of not more than 3 bar for about 2 h using a 1:1 weight ratio of steam: raw wood chips. The volatile pyrolysis products left the reactor via an exhaust pipe with a diameter of 25 mm (Fig. 6f) and were allowed to vent to the atmosphere after passing the condenser and liquid–gas separator. After steam activation at the desired temperature for 2 h, the wood stove and steam were turned off and the kiln was let to cool down overnight. The activated carbon materials were discharged from the kiln and ground for characterization.

### Characterization of eucalyptus wood chips and activated carbon

The sun dried eucalyptus wood chips and activated carbon materials were subjected to approximate analysis using standard methods^21–24^. The materials were also characterized by FTIR (Spectrum GX, Perkin Elmer), SEM–EDS (Mode LEO 1455 VP, LEO Electron Microscopy Ltd, England), Raman spectroscopy (Bruker, MultiRAM), XRD (PW 3040/60 X’Pert PRO Console, Philips, Netherlands), TGA (PW 3040/60 X’Pert PRO Console, Philips, Netherlands), and BET (Micromeritics TriStar II). Iodine and methylene number were measured for the activated carbon products. Finally, the percent yields of activated carbon materials obtained from steam activation at temperatures of 500 °C, 600 °C, and 700 °C were calculated.
